# Supplementary material for: Transcriptional Profiling of Whisker Follicles and of the Striatum in Methamphetamine Self-Administered Rats
Source: Int J Mol Sci. 2020 Nov 23;21(22):8856. doi: 10.3390/ijms21228856 (PMC7700365; doi:10.3390/ijms21228856)
Supplement: Supplementary file 1 [file ijms-21-08856-s001.pdf]

SUPPLEMENTAL INFORMATION

Transcriptional Profiling of Whisker Follicles and of the Striatum in Methamphetamine Self-Administered Rats

Won-Jun Jang [1,#], Taekwon Son [2,#], Sang-Hoon Song [1], In Soo Ryu [3], Sooyeun Lee [1,*] and Chul-Ho Jeong [1,*]

Supplementary Materials and Methods

Western blot analysis

The concentration of the proteins was determined using the BCA protein assay kit (Thermo Fisher Scientific, Waltham, MA USA). The proteins were separated by sodium dodecyl sulfate-polyacrylamide gel electrophoresis (SDS-PAGE) and transferred to PVDF membranes (Millipore Corp., Bedford, MA, USA). The membranes were blocked with blocking buffer containing 5% BSA in a mixture of Tris-buffered saline and Tween-20 (TBS-T), then washed three times for 10 min each with TBS-T. After washing, the membranes were probed with either primary antibodies against Per1 (sc-398890, 1:100), Arc (sc-17839, 1:100), JunB (sc-8051, 1:100), TH (sc-25269, 1:100) p-TH (#2791, 1:100), D1DR (sc-33660, 1:100), BDNF (ab108319, 1:100), and β-actin (#4970, 1:1000) for 18h at 4 ℃. The membranes were washed with TBS-T three times again and incubated with HRP-conjugated secondary antibody (1:5000 dilution, Thermo Fisher Scientific, Waltham, MA USA) under the same conditions as the primary antibodies for 2 h at room temperature. The protein bands were visualized using WesternBright™ ECL (advansta, San jose, CA, USA) and developed with LAS-3000 (Fuji, Japan). All primary antibodies were purchased from Santa Cruz Biotechnology (Dallas, TX, USA) except for the antibody against β-actin, p-TH (Cell Signaling Technology, Danvers, MA, USA) and BDNF (Abcam, Cambridge, MA, USA). Densitometric analysis was performed with ImageJ software. Data were normalized to the values yielded for β-actin.

Reverse transcription-polymerase chain reaction (RT-PCR) analysis

Complementary DNA (cDNA) was synthesized from 500ng total RNA using M-MLV reverse transcriptase (Promega, Madison, WI, USA). The PCR reaction was performed using ExTaq polymerase (TaKaRa, Japan). PCR conditions were optimized to primer or efficiency of target gene amplification. The primers for App were F- CTTGCACGACTATGGCATGC, R- ACACCTCTCGGACTACCTCC, Ddit4; F- CAGAGAAGAGGGCCTTGACC, R- ACCAGGGACCAAGGAAGAGT, β-actin; F-CCCATCTATGAGGGTTACGC, R-TTCTGCATCCTGTCAGCAAT. Amplified products were run on the 2% agarose gel and detected by LAS-3000 (Fuji, Japan). Densitometric analysis was performed with ImageJ software. Data were normalized to the values yielded for β-actin.

Statistical analysis

Western blotting and RT-PCR data are shown as mean ± SEM of four independent samples. Graph plotting and statistical analysis used GraphPad Prism Version 8.0 (GraphPad Software). Statistical

evaluation was performed by student t-test. *, $p < 0.05$ and **, $p < 0.01$, *** $p < 0.001$ were considered statistically significant.

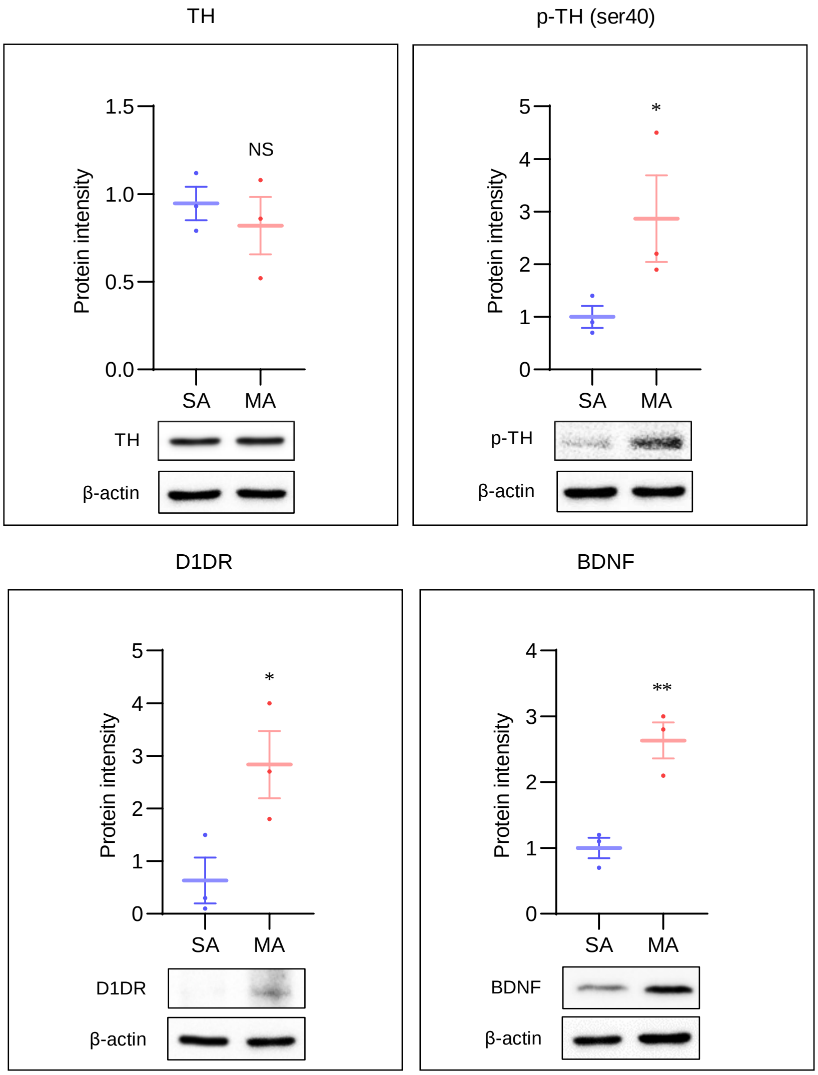

Figure S1. Protein expression levels of dopamine signaling in striatum.

Table S1. List of differentially expressed genes (87 genes) in striatum

**Up-regulated DEGs (73 genes)**					
Symbol	EntrezID	Description	FC	*p*-value	FDR
Tnnt2	24837	troponin T2, cardiac type	12.1	0.000	0.032
Cpxm2	293566	carboxypeptidase X (M14 family), member 2	11.6	0.000	0.006
Slco2a1	24546	solute carrier organic anion transporter family, member 2a1	5.8	0.000	0.029
Slc22a6	29509	solute carrier family 22 member 6	5.7	0.001	0.047
Nr4a3	58853	nuclear receptor subfamily 4, group A, member 3	5.6	0.000	0.006
Ccn5	29576	cellular communication network factor 5	4.7	0.000	0.011
Fosl2	25446	FOS like 2, AP-1 transcription factor subunit	4.6	0.000	0.006
Serpinf1	287526	serpin family F member 1	4.0	0.000	0.006
Alox15	81639	arachidonate 15-lipoxygenase	3.5	0.001	0.042
Alas2	25748	5'-aminolevulinate synthase 2	3.5	0.000	0.006
Nr4a1	79240	nuclear receptor subfamily 4, group A, member 1	3.4	0.000	0.006
Egr2	114090	early growth response 2	3.3	0.000	0.006
Hba-a2	360504	hemoglobin alpha, adult chain 2	3.1	0.000	0.006
LOC100134871	100134871	beta globin minor gene	3.0	0.000	0.006
Hbb	24440	hemoglobin subunit beta	3.0	0.000	0.006
Hbb-b1	361619	hemoglobin, beta adult major chain	2.8	0.000	0.006
Fmod	64507	fibromodulin	2.7	0.000	0.032
Tgm2	56083	transglutaminase 2	2.7	0.000	0.006
Slc13a4	503568	solute carrier family 13 member 4	2.6	0.001	0.040
Igfbp2	25662	insulin-like growth factor binding protein 2	2.6	0.000	0.006
Acta2	81633	actin, alpha 2, smooth muscle, aorta	2.5	0.000	0.006
Rn18s	100861533	18S ribosomal RNA	2.5	0.000	0.006
Cd74	25599	CD74 molecule	2.4	0.000	0.015
Arc	54323	activity-regulated cytoskeleton-associated protein	2.4	0.000	0.006
Egr4	25129	early growth response 4	2.4	0.000	0.006
Col1a1	29393	collagen type I alpha 1 chain	2.4	0.000	0.006
Efemp1	305604	EGF containing fibulin extracellular matrix protein 1	2.3	0.000	0.006
Col3a1	84032	collagen type III alpha 1 chain	2.3	0.000	0.019
Lrg1	367455	leucine-rich alpha-2-glycoprotein 1	2.3	0.000	0.011
Selenow	25545	selenoprotein W	2.2	0.000	0.006
Sult1a1	83783	sulfotransferase family 1A member 1	2.1	0.001	0.045
Fosb	100360880	FosB proto-oncogene, AP-1 transcription factor subunit	2.0	0.000	0.022
Junb	24517	JunB proto-oncogene, AP-1 transcription factor subunit	2.0	0.000	0.006
Fam107a	361018	family with sequence similarity 107, member A	2.0	0.000	0.006
Fxyd5	60338	FXYD domain-containing ion transport regulator 5	1.9	0.000	0.029
Mgp	25333	matrix Gla protein	1.9	0.000	0.026
Htra3	360959	HtrA serine peptidase 3	1.9	0.001	0.036
Igf2	24483	insulin-like growth factor 2	1.9	0.000	0.006
Hsd11b1	25116	hydroxysteroid 11-beta dehydrogenase 1	1.9	0.000	0.006
Tagln	25123	transgelin	1.8	0.000	0.034
Lyz2	25211	lysozyme 2	1.8	0.000	0.006
Alpl	25586	alkaline phosphatase, biomineralization associated	1.8	0.000	0.034
Cxcl14	306748	C-X-C motif chemokine ligand 14	1.8	0.000	0.006
Klf10	81813	Kruppel-like factor 10	1.7	0.001	0.047
RGD1564664	499839	similar to LOC387763 protein	1.7	0.000	0.006
Apln	58812	apelin	1.7	0.000	0.006
Slc25a25	246771	solute carrier family 25 member 25	1.7	0.000	0.006
Irs2	29376	insulin receptor substrate 2	1.7	0.000	0.006
Bgn	25181	biglycan	1.7	0.001	0.049
Per1	287422	period circadian regulator 1	1.6	0.000	0.011
Serpinh1	29345	serpin family H member 1	1.6	0.000	0.011
Tmem119	304581	transmembrane protein 119	1.6	0.000	0.011
Ddit4	140942	DNA-damage-inducible transcript 4	1.6	0.000	0.006
Rnase4	56759	ribonuclease A family member 4	1.6	0.000	0.006
Aifm3	303786	apoptosis inducing factor, mitochondria associated 3	1.6	0.000	0.006
Dusp1	114856	dual specificity phosphatase 1	1.6	0.000	0.029
Ier5	498256	immediate early response 5	1.6	0.000	0.034
Rem2	64626	RRAD and GEM like GTPase 2	1.6	0.000	0.006
Sod3	25352	superoxide dismutase 3	1.6	0.000	0.026
Htra1	65164	HtrA serine peptidase 1	1.6	0.000	0.006
Sgk1	29517	serum/glucocorticoid regulated kinase 1	1.6	0.000	0.006
Gjb6	84403	gap junction protein, beta 6	1.6	0.001	0.038
Fzd2	64512	frizzled class receptor 2	1.6	0.001	0.044
C1qa	298566	complement C1q A chain	1.6	0.000	0.006

Wfdc1	171112	WAP four-disulfide core domain 1	1.6	0.001	0.040
Wfs1	83725	wolframin ER transmembrane glycoprotein	1.6	0.001	0.049
Vim	81818	vimentin	1.6	0.001	0.042
Tmem132e	287564	transmembrane protein 132E	1.6	0.000	0.034
Cdc42ep4	303653	CDC42 effector protein 4	1.5	0.001	0.036
Fn1	25661	fibronectin 1	1.5	0.001	0.040
C1qb	29687	complement C1q B chain	1.5	0.001	0.036
Metrn	287151	meteorin, glial cell differentiation regulator	1.5	0.001	0.038
Pygm	24701	glycogen phosphorylase, muscle associated	1.5	0.001	0.047
**Down-regulated DEGs (14 genes)**					
Symbol	EntrezID	Description	FC	*p*-value	FDR
Oxt	25504	oxytocin/neurophysin 1 prepropeptide	-7.0	0.000	0.006
Krt86	407760	keratin 86	-5.4	0.001	0.042
Slc17a6	84487	solute carrier family 17 member 6	-4.7	0.000	0.006
Cbln1	498922	cerebellin 1 precursor	-4.1	0.000	0.022
Krt33a	303527	keratin 33A	-4.1	0.001	0.045
Slc17a7	116638	solute carrier family 17 member 7	-3.1	0.000	0.006
Miat	102552664	myocardial infarction associated transcript	-2.0	0.000	0.006
Hapln4	361129	hyaluronan and proteoglycan link protein 4	-1.9	0.000	0.006
Snhg11	362256	small nucleolar RNA host gene 11	-1.8	0.000	0.006
Rbm3	114488	RNA binding motif protein 3	-1.6	0.000	0.034
Gria2	29627	glutamate ionotropic receptor AMPA type subunit 2	-1.6	0.000	0.032
Gabra1	29705	gamma-aminobutyric acid type A receptor alpha1 subunit	-1.6	0.000	0.019
Pnisr	297942	PNN interacting serine and arginine rich protein	-1.6	0.000	0.006
Leng8	361506	leukocyte receptor cluster member 8	-1.5	0.001	0.040

DEGs, differentially expressed genes; FC, fold change; FDR, false discovery rate.

Table S2. List of differentially expressed genes (253 genes) in whisker follicle

**Up-regulated DEGs (71 genes)**					
Symbol	EntrezID	Description	FC	*p*-value	FDR
Selenow	25545	selenoprotein W	inf	0.000	0.006
Tnfrsf11b	25341	TNF receptor superfamily member 11B	3.4	0.001	0.036
Olfml3	310743	olfactomedin-like 3	2.6	0.001	0.036
LOC680428	680428	hypothetical protein LOC680428	2.4	0.000	0.019
Ka11	450226	type I keratin KA11	2.4	0.000	0.011
Uchl1	29545	ubiquitin C-terminal hydrolase L1	2.4	0.000	0.006
Krt36	287698	keratin 36	2.3	0.000	0.019
Eif3h	299899	eukaryotic translation initiation factor 3, subunit H	2.2	0.000	0.006
Pard6g	307237	par-6 family cell polarity regulator gamma	2.2	0.000	0.026
Ntrk2	25054	neurotrophic receptor tyrosine kinase 2	2.1	0.000	0.006
Wfs1	83725	wolframin ER transmembrane glycoprotein	2.1	0.000	0.011
Cavin1	287710	caveolae associated protein 1	2.1	0.001	0.040
Tm4sf4	116467	transmembrane 4 L six family member 4	2.1	0.000	0.006
March7	311059	membrane associated ring-CH-type finger 7	2.0	0.000	0.011
Dpp3	114591	dipeptidylpeptidase 3	2.0	0.001	0.038
Per1	287422	period circadian regulator 1	2.0	0.000	0.006
Ddr1	25678	discoidin domain receptor tyrosine kinase 1	2.0	0.000	0.011
Tspan14	306324	tetraspanin 14	2.0	0.000	0.006
Ivl	60583	involucrin	2.0	0.000	0.006
Krt75	300247	keratin 75	2.0	0.000	0.006
Hspa9	291671	heat shock protein family A member 9	1.9	0.001	0.042
Irf2bpl	314329	interferon regulatory factor 2 binding protein-like	1.9	0.000	0.015
Ubb	192255	ubiquitin B	1.9	0.000	0.006
Gsdmc	299908	gasdermin C	1.9	0.000	0.006
Ggt6	408206	gamma-glutamyl transferase 6	1.9	0.000	0.006
Atl2	298757	atlastin GTPase 2	1.9	0.000	0.032
Oga	154968	O-GlcNAcase	1.9	0.001	0.045
Uba2	308508	ubiquitin-like modifier activating enzyme 2	1.9	0.000	0.029
Sp6	363672	Sp6 transcription factor	1.8	0.000	0.032
Krtap19-5	685304	keratin associated protein 19-5	1.8	0.000	0.011
Dmkn	361548	dermokine	1.8	0.000	0.015
Krt82	366991	keratin 82	1.8	0.000	0.015
Arl6ip5	66028	ADP-ribosylation factor like GTPase 6 interacting protein 5	1.8	0.000	0.032
Smo	25273	smoothened, frizzled class receptor	1.8	0.001	0.047
Plekhm2	313667	pleckstrin homology and RUN domain containing M2	1.8	0.001	0.036
Ctnnb1	84353	catenin beta 1	1.8	0.001	0.040
Lrrc8e	304203	leucine rich repeat containing 8 VRAC subunit E	1.8	0.000	0.006
Cadm4	365216	cell adhesion molecule 4	1.8	0.000	0.015
Tpm4	24852	tropomyosin 4	1.8	0.001	0.040
Pdcd4	64031	programmed cell death 4	1.8	0.000	0.006
Endou	680317	endonuclease, poly(U)-specific	1.7	0.000	0.011
Akt1	24185	AKT serine/threonine kinase 1	1.7	0.000	0.011
Acy3	293653	aminoacylase 3	1.7	0.000	0.006
Slc34a2	84395	solute carrier family 34 member 2	1.7	0.000	0.006
Hnrnph2	308650	heterogeneous nuclear ribonucleoprotein H2	1.7	0.000	0.034
Serpinb13	304690	serpin family B member 13	1.7	0.000	0.006
Nub1	296731	negative regulator of ubiquitin-like proteins 1	1.7	0.000	0.006
Foxc2	171356	forkhead box C2	1.7	0.000	0.032
Bmp4	25296	bone morphogenetic protein 4	1.7	0.000	0.011
Gpcpd1	362219	glycerophosphocholine phosphodiesterase 1	1.7	0.000	0.015
Lrrc1	367113	leucine rich repeat containing 1	1.7	0.001	0.038
Syngr2	89815	synaptogyrin 2	1.6	0.000	0.019
Nfkbia	25493	NFKB inhibitor alpha	1.6	0.000	0.011
Ptprs	25529	protein tyrosine phosphatase, receptor type, S	1.6	0.001	0.045
Rnf19b	313806	ring finger protein 19B	1.6	0.000	0.034
Tagln	25123	transgelin	1.6	0.000	0.011
Alas1	65155	5'-aminolevulinate synthase 1	1.6	0.000	0.015
App	54226	amyloid beta precursor protein	1.6	0.000	0.029
Epgn	289515	epithelial mitogen	1.6	0.000	0.026
Id2	25587	inhibitor of DNA binding 2	1.6	0.000	0.011
Maff	366960	MAF bZIP transcription factor F	1.6	0.000	0.032
Ddit4	140942	DNA-damage-inducible transcript 4	1.6	0.000	0.006
Clpx	300786	caseinolytic mitochondrial matrix peptidase chaperone subunit	1.6	0.000	0.026
Gsg1	312793	germ cell associated 1	1.6	0.001	0.036

Mbtps1	89842	membrane-bound transcription factor peptidase, site 1	1.6	0.001	0.044
Tars	294810	threonyl-tRNA synthetase	1.5	0.001	0.042
Ror2	306782	receptor tyrosine kinase-like orphan receptor 2	1.5	0.001	0.047
Eml2	192360	EMAP like 2	1.5	0.000	0.034
Ap2a1	308578	adaptor related protein complex 2 subunit alpha 1	1.5	0.001	0.045
Dhcr7	64191	7-dehydrocholesterol reductase	1.5	0.000	0.034
Tmem65	500874	transmembrane protein 65	1.5	0.000	0.034
**Down-regulated DEGs (182 genes)**					
Symbol	EntrezID	Description	FC	*p*_value	FDR
Mrpl30	301352	mitochondrial ribosomal protein L30	-inf	0.000	0.006
Plp1	24943	proteolipid protein 1	-5.6	0.000	0.006
Rps9	81772	ribosomal protein S9	-3.9	0.000	0.006
Mbp	24547	myelin basic protein	-2.6	0.000	0.006
Rbp1	25056	retinol binding protein 1	-2.4	0.000	0.006
Stfa3	288075	stefin A3	-2.4	0.000	0.006
LOC100909539	100909539	uncharacterized LOC100909539	-2.4	0.000	0.022
Rps24	81776	ribosomal protein S24	-2.4	0.000	0.006
Tmem256	287442	transmembrane protein 256	-2.3	0.000	0.006
Il17b	116472	interleukin 17B	-2.3	0.000	0.006
Rps29	25348	ribosomal protein S29	-2.3	0.000	0.006
Il36a	296541	interleukin 36, alpha	-2.3	0.000	0.006
Rps27a	100912032	ribosomal protein S27a	-2.3	0.000	0.006
Rps17	29286	ribosomal protein S17	-2.2	0.000	0.006
S100a9	94195	S100 calcium binding protein A9	-2.2	0.001	0.045
Rps10	81773	ribosomal protein S10	-2.2	0.000	0.006
Sec61g	689134	Sec61 translocon gamma subunit	-2.2	0.000	0.006
Rpl22l1	361923	ribosomal protein L22 like 1	-2.1	0.000	0.006
Atp5f1e	245958	ATP synthase F1 subunit epsilon	-2.1	0.000	0.006
Rps27	94266	ribosomal protein S27	-2.1	0.000	0.006
Rpl13a	317646	ribosomal protein L13A	-2.1	0.000	0.006
Rpl13	81765	ribosomal protein L13	-2.1	0.000	0.006
Ifi27l2b	299269	interferon, alpha-inducible protein 27 like 2B	-2.0	0.000	0.006
Lce1m	688413	late cornified envelope 1M	-2.0	0.000	0.006
Npw	259224	neuropeptide W	-2.0	0.000	0.006
RGD1565641	499567	RGD1565641	-2.0	0.000	0.006
Gmfg	113940	glia maturation factor, gamma	-2.0	0.000	0.006
Rbp2	24710	retinol binding protein 2	-2.0	0.000	0.006
Csde1	117180	cold shock domain containing E1	-1.9	0.000	0.006
Rplp1	140661	ribosomal protein lateral stalk subunit P1	-1.9	0.001	0.045
Atp5me	140608	ATP synthase membrane subunit e	-1.9	0.000	0.006
Hbb	24440	hemoglobin subunit beta	-1.9	0.001	0.036
Cstb	25308	cystatin B	-1.9	0.000	0.019
Nrtn	84423	neurturin	-1.9	0.000	0.026
Lce1f	499663	late cornified envelope 1F	-1.9	0.000	0.006
Rpl27a	293418	ribosomal protein L27a	-1.9	0.000	0.006
Rpl22	81768	ribosomal protein L22	-1.9	0.000	0.006
Mrps33	296995	mitochondrial ribosomal protein S33	-1.9	0.000	0.006
Tra2b	117259	transformer 2 beta homolog	-1.9	0.000	0.006
Elavl1	363854	ELAV like RNA binding protein 1	-1.9	0.000	0.006
Chchd1	361005	coiled-coil-helix-coiled-coil-helix domain containing 1	-1.9	0.000	0.006
Uqcc2	361805	ubiquinol-cytochrome c reductase complex assembly factor 2	-1.9	0.000	0.006
RGD1563941	500993	similar to hypothetical protein FLJ20010	-1.9	0.000	0.011
Luc7l3	360602	LUC7-like 3 pre-mRNA splicing factor	-1.9	0.000	0.006
Tmem160	292654	transmembrane protein 160	-1.9	0.000	0.006
Atp5mg	300677	ATP synthase membrane subunit g	-1.9	0.000	0.006
Atp5mc1	29754	ATP synthase membrane subunit c locus 1	-1.8	0.000	0.006
Stmn4	79423	stathmin 4	-1.8	0.000	0.006
Eif3el1	299872	eukaryotic translation initiation factor 3, subunit E-like 1	-1.8	0.000	0.019
Rsrp1	362626	arginine and serine rich protein 1	-1.8	0.000	0.006
Rpl37a	100361520	ribosomal protein L37a	-1.8	0.000	0.032
S100a1	295214	S100 calcium binding protein A1	-1.8	0.000	0.015
RGD1310587	360894	similar to hypothetical protein FLJ14146	-1.8	0.001	0.049
Rpl28	64638	ribosomal protein L28	-1.8	0.001	0.049
Atp5mc3	114630	ATP synthase membrane subunit c locus 3	-1.8	0.000	0.006
Trir	288920	telomerase RNA component interacting RNase	-1.8	0.000	0.006
Sdf2l1	680945	stromal cell-derived factor 2-like 1	-1.8	0.000	0.022
Ubc	50522	ubiquitin C	-1.8	0.000	0.006
Smim22	360478	small integral membrane protein 22	-1.8	0.001	0.036

Jpt1	287828	Jupiter microtubule associated homolog 1	-1.8	0.000	0.006
Uqcrb	362897	ubiquinol-cytochrome c reductase binding protein	-1.8	0.000	0.006
Polr2k	100361574	RNA polymerase II subunit K	-1.8	0.000	0.006
Mrps18c	289469	mitochondrial ribosomal protein S18C	-1.8	0.000	0.011
LOC100911177	100911177	uncharacterized LOC100911177	-1.8	0.000	0.006
Fxyd4	64190	FXYD domain-containing ion transport regulator 4	-1.8	0.000	0.006
MGC105649	302884	hypothetical LOC302884	-1.8	0.000	0.006
Pfdn5	300257	prefoldin subunit 5	-1.7	0.000	0.006
Rps15a	117053	ribosomal protein S15a	-1.7	0.000	0.006
LOC100912041	100912041	uncharacterized LOC100912041	-1.7	0.000	0.006
Arpp19	60336	cAMP-regulated phosphoprotein 19	-1.7	0.000	0.006
Tmsb10	50665	thymosin, beta 10	-1.7	0.000	0.006
Tomm7	685620	translocase of outer mitochondrial membrane 7	-1.7	0.000	0.006
Cebpb	24253	CCAAT/enhancer binding protein beta	-1.7	0.000	0.006
Nme1	191575	NME/NM23 nucleoside diphosphate kinase 1	-1.7	0.000	0.006
Tardbp	298648	TAR DNA binding protein	-1.7	0.000	0.006
Tbca	366995	tubulin folding cofactor A	-1.7	0.000	0.006
Anapc11	498030	anaphase promoting complex subunit 11	-1.7	0.000	0.006
Lsm7	362829	LSM7 homolog, U6 small nuclear RNA and mRNA degradation associated	-1.7	0.000	0.011
Dnlz	296587	DNL-type zinc finger	-1.7	0.000	0.006
Smim26	296207	small integral membrane protein 26	-1.7	0.000	0.015
Elob	81807	elongin B	-1.7	0.000	0.011
Serpina11	362774	serpin family A member 11	-1.7	0.000	0.006
Naa38	287429	N(alpha)-acetyltransferase 38, NatC auxiliary subunit	-1.7	0.000	0.015
Prdx1	117254	peroxiredoxin 1	-1.7	0.000	0.022
Sf3b5	680891	splicing factor 3b, subunit 5	-1.7	0.000	0.006
Il18	29197	interleukin 18	-1.7	0.000	0.011
Ndufa5	25488	NADH:ubiquinone oxidoreductase subunit A5	-1.7	0.000	0.006
Defb1	83687	defensin beta 1	-1.7	0.000	0.006
Lsm3	297455	LSM3 homolog, U6 small nuclear RNA and mRNA degradation associated	-1.7	0.000	0.006
Lce1l	686125	late cornified envelope 1L	-1.7	0.000	0.019
Gpx4	29328	glutathione peroxidase 4	-1.7	0.000	0.006
Ogt	26295	O-linked N-acetylglucosamine (GlcNAc) transferase	-1.7	0.000	0.006
Scand1	362252	SCAN domain-containing 1	-1.7	0.000	0.006
Arl4d	303559	ADP-ribosylation factor like GTPase 4D	-1.7	0.000	0.006
Creld2	362978	cysteine-rich with EGF-like domains 2	-1.7	0.000	0.019
Ndufa2	291660	NADH:ubiquinone oxidoreductase subunit A2	-1.7	0.000	0.006
Nr2c2ap	361128	nuclear receptor 2C2-associated protein	-1.7	0.000	0.006
Uqcrq	497902	ubiquinol-cytochrome c reductase, complex III subunit VII	-1.7	0.000	0.019
Cox20	289278	cytochrome c oxidase assembly factor COX20	-1.7	0.000	0.034
Snrpd2	680309	small nuclear ribonucleoprotein D2 polypeptide	-1.7	0.000	0.006
Rps3a	29288	ribosomal protein S3a	-1.7	0.000	0.015
Gapdh	24383	glyceraldehyde-3-phosphate dehydrogenase	-1.7	0.000	0.011
Sod1	24786	superoxide dismutase 1	-1.7	0.000	0.006
Mrps35	297727	mitochondrial ribosomal protein S35	-1.7	0.000	0.006
Psmb6	29666	proteasome subunit beta 6	-1.7	0.000	0.026
Uqcrh	366448	ubiquinol-cytochrome c reductase hinge protein	-1.7	0.000	0.011
Sub1	192269	SUB1 homolog, transcriptional regulator	-1.7	0.000	0.006
S100a8	116547	S100 calcium binding protein A8	-1.6	0.001	0.047
Rpl34	362041	ribosomal protein L34	-1.6	0.001	0.038
Ube2s	292588	ubiquitin-conjugating enzyme E2S	-1.6	0.000	0.006
Magoh	298385	mago homolog, exon junction complex subunit	-1.6	0.000	0.006
Rps27l	681429	ribosomal protein S27-like	-1.6	0.000	0.006
Atp5pd	641434	ATP synthase peripheral stalk subunit d	-1.6	0.000	0.034
Serf2	502663	small EDRK-rich factor 2	-1.6	0.001	0.042
Vamp8	83730	vesicle-associated membrane protein 8	-1.6	0.000	0.006
Ndufb2	362344	NADH:ubiquinone oxidoreductase subunit B2	-1.6	0.000	0.006
S100a10	81778	S100 calcium binding protein A10	-1.6	0.001	0.047
Gstm5	64352	glutathione S-transferase, mu 5	-1.6	0.000	0.006
Bloc1s1	288785	biogenesis of lysosomal organelles complex-1, subunit 1	-1.6	0.000	0.019
Mrpl41	296551	mitochondrial ribosomal protein L41	-1.6	0.000	0.026
Ndufa7	299643	NADH:ubiquinone oxidoreductase subunit A7	-1.6	0.000	0.019
Arglu1	290912	arginine and glutamate rich 1	-1.6	0.000	0.011
Cltb	116561	clathrin, light chain B	-1.6	0.001	0.038
Rpl36al	81769	ribosomal protein L36a-like	-1.6	0.000	0.019
Pfdn1	361310	prefoldin subunit 1	-1.6	0.000	0.011
RT1-A2	24974	RT1 class Ia, locus A2	-1.6	0.001	0.042
Mif	81683	macrophage migration inhibitory factor	-1.6	0.000	0.022

Rps23	124323	ribosomal protein S23	-1.6	0.000	0.029
Mrpl55	287356	mitochondrial ribosomal protein L55	-1.6	0.000	0.006
Exosc7	316098	exosome component 7	-1.6	0.000	0.011
Eif3k	292762	eukaryotic translation initiation factor 3, subunit K	-1.6	0.000	0.019
Psmg4	689623	proteasome assembly chaperone 4	-1.6	0.000	0.019
Use1	290627	unconventional SNARE in the ER 1	-1.6	0.000	0.015
Mrps21	689432	mitochondrial ribosomal protein S21	-1.6	0.000	0.019
Atp6v0b	298451	ATPase H+ transporting V0 subunit B	-1.6	0.000	0.026
Mrpl23	64360	mitochondrial ribosomal protein L23	-1.6	0.000	0.011
Pop5	117241	POP5 homolog, ribonuclease P/MRP subunit	-1.6	0.000	0.011
Nop10	691534	NOP10 ribonucleoprotein	-1.6	0.000	0.011
Gar1	499709	GAR1 ribonucleoprotein	-1.6	0.001	0.040
Cmss1	288176	cms1 ribosomal small subunit homolog	-1.6	0.000	0.026
Cox17	89786	cytochrome c oxidase copper chaperone COX17	-1.6	0.000	0.029
Micos10	362641	mitochondrial contact site and cristae organizing system subunit 10	-1.6	0.000	0.019
Sept7	64551	septin 7	-1.6	0.000	0.006
Ciao2b	680987	cytosolic iron-sulfur assembly component 2B	-1.6	0.001	0.044
Psenen	292788	presenilin enhancer gamma secretase subunit	-1.6	0.001	0.042
Churc1	299154	churchill domain containing 1	-1.6	0.000	0.006
Timm13	252928	translocase of inner mitochondrial membrane 13	-1.6	0.001	0.038
Fxn	499335	frataxin	-1.6	0.001	0.049
Fbxl15	309453	F-box and leucine-rich repeat protein 15	-1.6	0.001	0.047
Nphp1	296136	nephrocystin 1	-1.6	0.000	0.019
Hint2	313491	histidine triad nucleotide binding protein 2	-1.6	0.001	0.045
Fis1	288584	fission, mitochondrial 1	-1.6	0.001	0.036
Mrpl52	361037	mitochondrial ribosomal protein L52	-1.6	0.001	0.036
Tmem14c	171432	transmembrane protein 14C	-1.6	0.001	0.045
Arl4a	29308	ADP-ribosylation factor like GTPase 4A	-1.6	0.000	0.022
Cd99	652929	CD99 molecule (Xg blood group)	-1.5	0.001	0.044
Cnih4	289324	cornichon family AMPA receptor auxiliary protein 4	-1.5	0.000	0.011
Bola1	365875	bolA family member 1	-1.5	0.001	0.049
Pgp	287115	phosphoglycolate phosphatase	-1.5	0.001	0.044
Srsf3	361814	serine and arginine rich splicing factor 3	-1.5	0.000	0.029
Sat1	302642	spermidine/spermine N1-acetyl transferase 1	-1.5	0.001	0.036
Sec61b	298068	Sec61 translocon beta subunit	-1.5	0.000	0.029
Arpc5l	296710	actin related protein 2/3 complex, subunit 5-like	-1.5	0.000	0.034
Cox7b	303393	cytochrome c oxidase subunit 7B	-1.5	0.001	0.040
Atp5pf	94271	ATP synthase peripheral stalk subunit F6	-1.5	0.000	0.034
Ndufb5	294964	NADH:ubiquinone oxidoreductase subunit B5	-1.5	0.001	0.038
Polr2g	117017	RNA polymerase II subunit G	-1.5	0.001	0.038
Krtcap2	295243	keratinocyte associated protein 2	-1.5	0.001	0.040
Prcp	293118	prolylcarboxypeptidase	-1.5	0.001	0.045
Glrx5	362776	glutaredoxin 5	-1.5	0.001	0.038
Fkbp2	293702	FKBP prolyl isomerase 2	-1.5	0.001	0.038
Nudt14	299346	nudix hydrolase 14	-1.5	0.001	0.045
Abracl	685045	ABRA C-terminal like	-1.5	0.001	0.036
Polr1d	360762	RNA polymerase I and III subunit D	-1.5	0.001	0.044
Tuft1	365864	tuftelin 1	-1.5	0.001	0.044
Cdk2ap2	688405	cyclin-dependent kinase 2 associated protein 2	-1.5	0.001	0.047
Mrps16	688912	mitochondrial ribosomal protein S16	-1.5	0.001	0.049
Mrpl54	299628	mitochondrial ribosomal protein L54	-1.5	0.001	0.047
Ostc	362040	oligosaccharyltransferase complex non-catalytic subunit	-1.5	0.001	0.038
Rpl10a	81729	ribosomal protein L10A	-1.5	0.001	0.038
Xbp1	289754	X-box binding protein 1	-1.5	0.001	0.045
Serpinb1a	291091	serpin family B member 1A	-1.5	0.001	0.045

DEGs, differentially expressed genes; FC, fold change; FDR, false discovery rate.

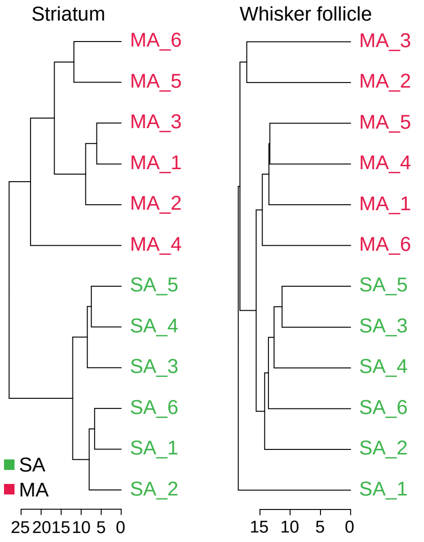

Figure S2. The hierarchical cluster analysis of DEGs in the striatum and whisker follicle.

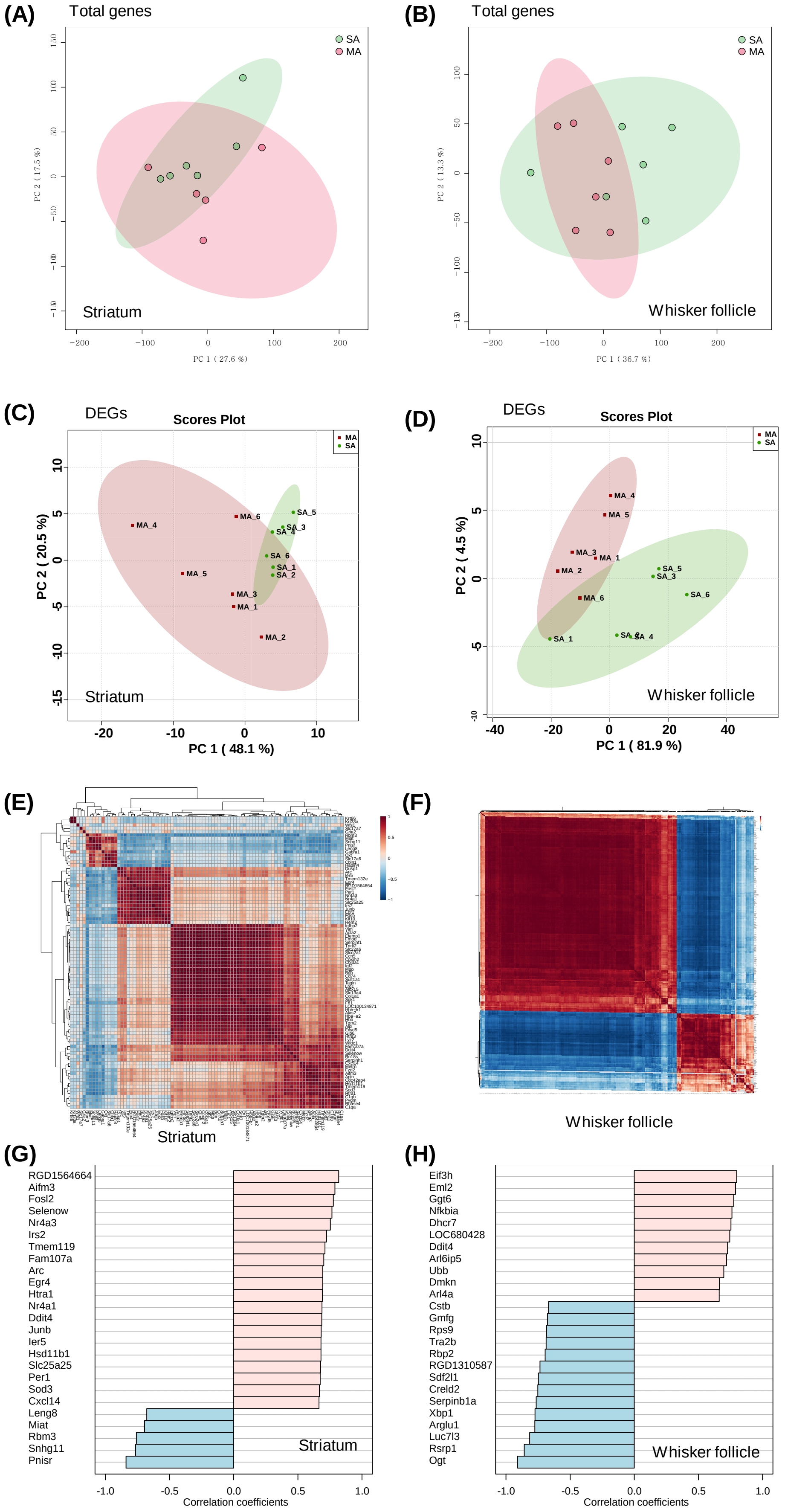

Figure S3. PCA and correlation pattern analysis of DEGs in the striatum and whisker follicle. (A-B) The 2D score plot of PCA of total genes from the striatum or whisker follicle. (C-D) The 2D score plot of PCA of DEGs from the striatum or whisker follicle. (E-F) The correlation matrix between different DEGs of the striatum or whisker follicle (the distance measure is Pearson's *r*). (G-H) Lists of the top 25 genes were obtained by correlation pattern searching under the two conditions, SA and MA (the distance measure is Pearson's *r*).

Table S3. Correlation coefficient list of DEGs in striatum

Symbol	EntrezID	Description	FC	*p*_value	FDR	correlation r
RGD1564664	499839	similar to LOC387763 protein	1.7	0.000	0.006	0.79
Aifm3	303786	apoptosis inducing factor, mitochondria associated 3	1.6	0.000	0.006	0.73
Fosl2	25446	FOS like 2, AP-1 transcription factor subunit	4.6	0.000	0.006	0.72
Selenow	25545	selenoprotein W	2.2	0.000	0.006	0.71
Nr4a3	58853	nuclear receptor subfamily 4, group A, member 3	5.6	0.000	0.006	0.71
Irs2	29376	insulin receptor substrate 2	1.7	0.000	0.006	0.69
Tmem119	304581	transmembrane protein 119	1.6	0.000	0.011	0.69
Fam107a	361018	family with sequence similarity 107, member A	2.0	0.000	0.006	0.69
Arc	54323	activity-regulated cytoskeleton-associated protein	2.4	0.000	0.006	0.69
Egr4	25129	early growth response 4	2.4	0.000	0.006	0.68
Htra1	65164	HtrA serine peptidase 1	1.6	0.000	0.006	0.67
Nr4a1	79240	nuclear receptor subfamily 4, group A, member 1	3.4	0.000	0.006	0.67
Ddit4	140942	DNA-damage-inducible transcript 4	1.6	0.000	0.006	0.65
Junb	24517	JunB proto-oncogene, AP-1 transcription factor subunit	2.0	0.000	0.006	0.65
Ier5	498256	immediate early response 5	1.6	0.000	0.034	0.65
Hsd11b1	25116	hydroxysteroid 11-beta dehydrogenase 1	1.9	0.000	0.006	0.65
Slc25a25	246771	solute carrier family 25 member 25	1.7	0.000	0.006	0.65
Per1	287422	period circadian regulator 1	1.6	0.000	0.011	0.64
Sod3	25352	superoxide dismutase 3	1.6	0.000	0.026	0.64
Cxcl14	306748	C-X-C motif chemokine ligand 14	1.8	0.000	0.006	0.63
Serpinh1	29345	serpin family H member 1	1.6	0.000	0.011	0.63
Klf10	81813	Kruppel-like factor 10	1.7	0.001	0.047	0.63
Apln	58812	apelin	1.7	0.000	0.006	0.62
Pygm	24701	glycogen phosphorylase, muscle associated	1.5	0.001	0.047	0.62
Htra3	360959	HtrA serine peptidase 3	1.9	0.001	0.036	0.61
Leng8	361506	leukocyte receptor cluster member 8	-1.5	0.001	0.040	-0.67
Miat	102552664	myocardial infarction associated transcript	-2.0	0.000	0.006	-0.70
Rbm3	114488	RNA binding motif protein 3	-1.6	0.000	0.034	-0.71
Snhg11	362256	small nucleolar RNA host gene 11	-1.8	0.000	0.006	-0.72
Pnisr	297942	PNN interacting serine and arginine rich protein	-1.6	0.000	0.006	-0.81

Table S4. Correlation coefficient list of DEGs in whisker follicle

Symbol	EntrezID	Description	FC	*p*_value	FDR	Correlation r
Ogt	26295	O-linked N-acetylglucosamine (GlcNAc) transferase	-1.7	0.000	0.006	-0.89
Rsrp1	362626	arginine and serine rich protein 1	-1.8	0.000	0.006	-0.87
Luc7l3	360602	LUC7-like 3 pre-mRNA splicing factor	-1.9	0.000	0.006	-0.84
Arglu1	290912	arginine and glutamate rich 1	-1.6	0.000	0.011	-0.78
Xbp1	289754	X-box binding protein 1	-1.5	0.001	0.045	-0.77
Serpinb1a	291091	serpin family B member 1A	-1.5	0.001	0.045	-0.76
Creld2	362978	cysteine-rich with EGF-like domains 2	-1.7	0.000	0.019	-0.75
Sdf2l1	680945	stromal cell-derived factor 2-like 1	-1.8	0.000	0.022	-0.74
RGD1310587	360894	similar to hypothetical protein FLJ14146	-1.8	0.001	0.049	-0.73
Rbp2	24710	retinol binding protein 2	-2.0	0.000	0.006	-0.68
Tra2b	117259	transformer 2 beta homolog	-1.9	0.000	0.006	-0.68
Rps9	81772	ribosomal protein S9	-3.9	0.000	0.006	-0.67
Gmfg	113940	glia maturation factor, gamma	-2.0	0.000	0.006	-0.67
Cstb	25308	cystatin B	-1.9	0.000	0.019	-0.66
Arl4a	29308	ADP-ribosylation factor like GTPase 4A	-1.6	0.000	0.022	-0.66
Rps27a	100912032	ribosomal protein S27a	-2.3	0.000	0.006	-0.66
Rpl27a	293418	ribosomal protein L27a	-1.9	0.000	0.006	-0.66
Tardbp	298648	TAR DNA binding protein	-1.7	0.000	0.006	-0.66
Defb1	83687	defensin beta 1	-1.7	0.000	0.006	-0.64
Lce1m	688413	late cornified envelope 1M	-2.0	0.000	0.006	-0.64
Il36a	296541	interleukin 36, alpha	-2.3	0.000	0.006	-0.64
Magoh	298385	mago homolog, exon junction complex subunit	-1.6	0.000	0.006	-0.64
MGC105649	302884	hypothetical LOC302884	-1.8	0.000	0.006	-0.64
Sat1	302642	spermidine/spermine N1-acetyl transferase 1	-1.5	0.001	0.036	-0.64
Polr2k	100361574	RNA polymerase II subunit K	-1.8	0.000	0.006	-0.63
RGD1565641	499567	RGD1565641	-2.0	0.000	0.006	-0.63
Ciao2b	680987	cytosolic iron-sulfur assembly component 2B	-1.6	0.001	0.044	-0.63
Atp5mg	300677	ATP synthase membrane subunit g	-1.9	0.000	0.006	-0.63
Pgp	287115	phosphoglycolate phosphatase	-1.5	0.001	0.044	-0.63
Rbp1	25056	retinol binding protein 1	-2.4	0.000	0.006	-0.63
Cox20	289278	cytochrome c oxidase assembly factor COX20	-1.7	0.000	0.034	-0.63
Cnih4	289324	cornichon family AMPA receptor auxiliary protein 4	-1.5	0.000	0.011	-0.63
Elavl1	363854	ELAV like RNA binding protein 1	-1.9	0.000	0.006	-0.63
Rps24	81776	ribosomal protein S24	-2.4	0.000	0.006	-0.62
Mrps33	296995	mitochondrial ribosomal protein S33	-1.9	0.000	0.006	-0.62
Arl4d	303559	ADP-ribosylation factor like GTPase 4D	-1.7	0.000	0.006	-0.62
Tmem256	287442	transmembrane protein 256	-2.3	0.000	0.006	-0.62
Tbca	366995	tubulin folding cofactor A	-1.7	0.000	0.006	-0.61
Ifi27l2b	299269	interferon, alpha-inducible protein 27 like 2B	-2.0	0.000	0.006	-0.61
Rpl22l1	361923	ribosomal protein L22 like 1	-2.1	0.000	0.006	-0.61
LOC100912041	100912041	uncharacterized LOC100912041	-1.7	0.000	0.006	-0.61
Mrpl41	296551	mitochondrial ribosomal protein L41	-1.6	0.000	0.026	-0.61
Lce1f	499663	late cornified envelope 1F	-1.9	0.000	0.006	-0.61
Rps27	94266	ribosomal protein S27	-2.1	0.000	0.006	-0.60
Atp5mc3	114630	ATP synthase membrane subunit c locus 3	-1.8	0.000	0.006	-0.60
Smim26	296207	small integral membrane protein 26	-1.7	0.000	0.015	-0.60
Pfdn5	300257	prefoldin subunit 5	-1.7	0.000	0.006	-0.60
Rps17	29286	ribosomal protein S17	-2.2	0.000	0.006	-0.60
App	54226	amyloid beta precursor protein	1.6	0.000	0.029	0.60
Acy3	293653	aminoacylase 3	1.7	0.000	0.006	0.60
Per1	287422	period circadian regulator 1	2.0	0.000	0.006	0.60
Wfs1	83725	wolframin ER transmembrane glycoprotein	2.1	0.000	0.011	0.60
Gpcpd1	362219	glycerophosphocholine phosphodiesterase 1	1.7	0.000	0.015	0.61
Cadm4	365216	cell adhesion molecule 4	1.8	0.000	0.015	0.61
Krt75	300247	keratin 75	1.9	0.000	0.006	0.61
Tm4sf4	116467	transmembrane 4 L six family member 4	2.1	0.000	0.006	0.62
Ivl	60583	involucrin	1.9	0.000	0.006	0.62
Syngr2	89815	synaptogyrin 2	1.6	0.000	0.019	0.63
Nub1	296731	negative regulator of ubiquitin-like proteins 1	1.7	0.000	0.006	0.64
Tmem65	500874	transmembrane protein 65	1.5	0.000	0.034	0.65
Serpinb13	304690	serpin family B member 13	1.7	0.000	0.006	0.66
Lrrc8e	304203	leucine rich repeat containing 8 VRAC subunit E	1.8	0.000	0.006	0.66
Dmkn	361548	dermokine	1.8	0.000	0.015	0.68
Ubb	192255	ubiquitin B	1.9	0.000	0.006	0.69
Arl6ip5	66028	ADP-ribosylation factor like GTPase 6 interacting protein 5	1.8	0.000	0.032	0.72
Ddit4	140942	DNA-damage-inducible transcript 4	1.6	0.000	0.006	0.73
LOC680428	680428	hypothetical protein LOC680428	2.4	0.000	0.019	0.74
Dhcr7	64191	7-dehydrocholesterol reductase	1.5	0.000	0.034	0.74
Nfkbia	25493	NFKB inhibitor alpha	1.6	0.000	0.011	0.74
Ggt6	408206	gamma-glutamyl transferase 6	1.9	0.000	0.006	0.75
Eml2	192360	EMAP like 2	1.5	0.000	0.034	0.77
Eif3h	299899	eukaryotic translation initiation factor 3, subunit H	2.2	0.000	0.006	0.82

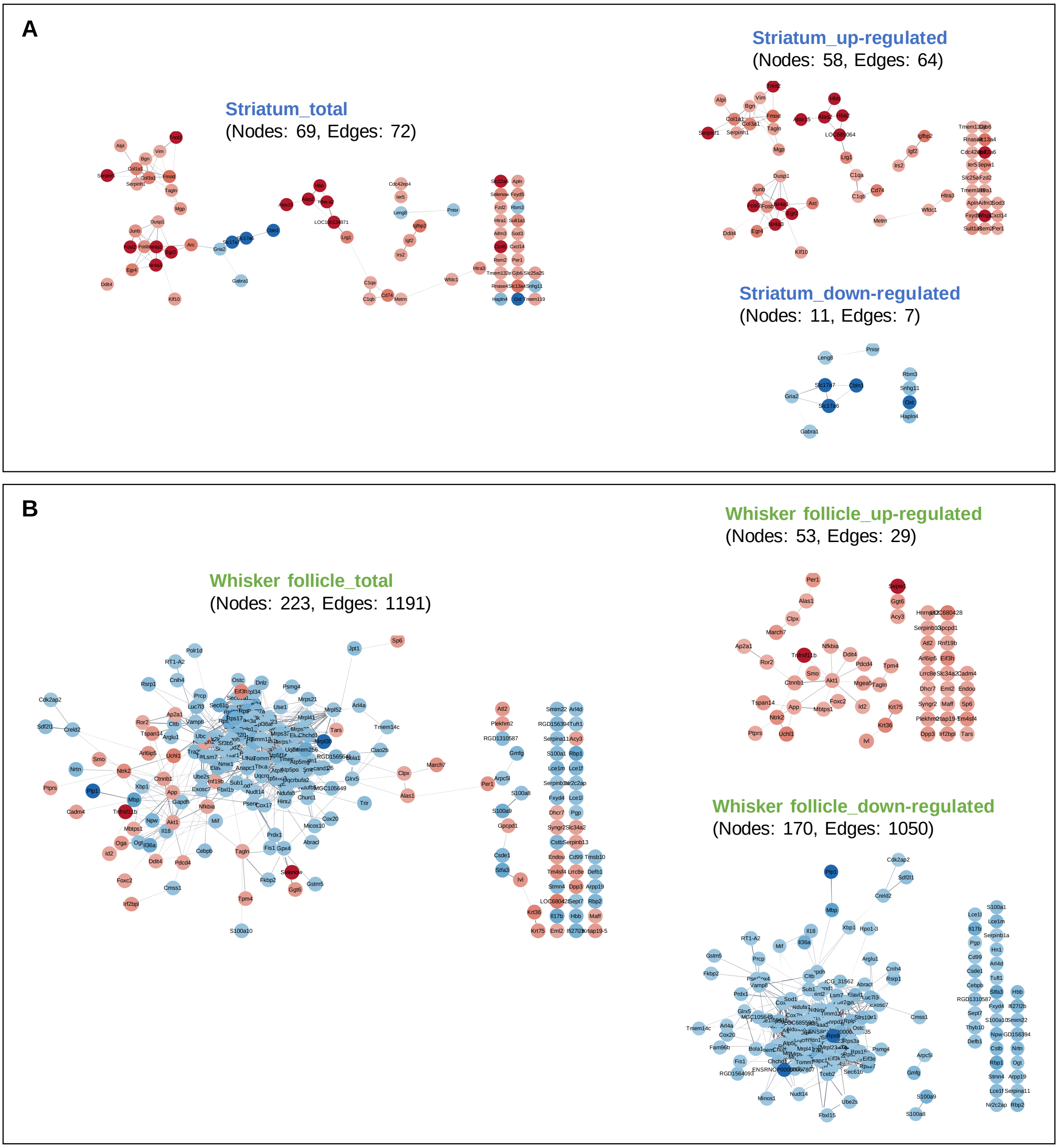

Figure S4. Construction of protein-protein interaction (PPI) networks from total, up- and down- regulated DEGs in the striatum and whisker follicle. The putative PPI networks of DEGs were visualised via the STRING application (confidence [score] cut-off = 0.4) in the Cytoscape software (v3.7.1). Red nodes denote up-regulated genes, and blue nodes indicate down-regulated genes. The intensity of each node colour means the fold change. (A) The PPI networks from total, up- and down-regulated DEGs of striatum. (B) The total, up- and down-regulated PPI networks from DEGs of whisker follicle.

Table S5. Functional enrichment analysis in PPI networks of the striatum

Total PPI Network

ID	Term	*p* -value	Ratio (%)	Count
**Biological Process**				
GO:0009612	response to mechanical stimulus	0.000	11.59	8
GO:0051591	response to cAMP	0.000	10.14	7
GO:0032355	response to estradiol	0.000	13.04	9
GO:0051384	response to glucocorticoid	0.000	10.14	7
GO:0007565	female pregnancy	0.000	8.70	6
GO:0001558	regulation of cell growth	0.000	7.25	5
GO:0042542	response to hydrogen peroxide	0.000	7.25	5
GO:0009416	response to light stimulus	0.000	5.80	4
GO:0030199	collagen fibril organization	0.000	5.80	4
GO:0001649	osteoblast differentiation	0.001	7.25	5
GO:0007568	aging	0.001	10.14	7
GO:0042060	wound healing	0.001	7.25	5
GO:0043434	response to peptide hormone	0.001	7.25	5
GO:0032870	cellular response to hormone stimulus	0.001	5.80	4
GO:0032570	response to progesterone	0.001	5.80	4
GO:0015671	oxygen transport	0.002	4.35	3
GO:0045444	fat cell differentiation	0.003	5.80	4
GO:0001503	ossification	0.005	5.80	4
GO:0045776	negative regulation of blood pressure	0.009	4.35	3
GO:0044344	cellular response to fibroblast growth factor stimulus	0.010	4.35	3
GO:0042493	response to drug	0.012	10.14	7
GO:0006366	transcription from RNA polymerase II promoter	0.014	8.70	6
GO:0071376	cellular response to corticotropin-releasing hormone stimulus	0.014	2.90	2
GO:0009314	response to radiation	0.015	4.35	3
GO:0071277	cellular response to calcium ion	0.016	4.35	3
GO:0048545	response to steroid hormone	0.020	4.35	3
GO:0032964	collagen biosynthetic process	0.021	2.90	2
GO:0007420	brain development	0.023	7.25	5
GO:0048839	inner ear development	0.024	4.35	3
GO:0061469	regulation of type B pancreatic cell proliferation	0.025	2.90	2
GO:0045944	positive regulation of transcription from RNA polymerase II promoter	0.030	13.04	9
GO:0032526	response to retinoic acid	0.031	4.35	3
GO:0051592	response to calcium ion	0.037	4.35	3
GO:0007267	cell-cell signaling	0.037	4.35	3
GO:0071333	cellular response to glucose stimulus	0.041	4.35	3
GO:0030431	sleep	0.042	2.90	2
GO:0014823	response to activity	0.043	4.35	3
GO:0042756	drinking behavior	0.049	2.90	2
GO:0034097	response to cytokine	0.049	4.35	3
**Cellular Component**				
GO:0031012	extracellular matrix	0.000	13.04	9
GO:0005615	extracellular space	0.000	23.19	16
GO:0005578	proteinaceous extracellular matrix	0.000	10.14	7
GO:0005581	collagen trimer	0.001	5.80	4
GO:0005833	hemoglobin complex	0.001	4.35	3
GO:0070062	extracellular exosome	0.001	28.99	20
GO:0030054	cell junction	0.003	10.14	7
GO:0005576	extracellular region	0.003	13.04	9
GO:0031838	haptoglobin-hemoglobin complex	0.014	2.90	2
GO:0030672	synaptic vesicle membrane	0.018	4.35	3
GO:0045211	postsynaptic membrane	0.032	5.80	4
**Molecular Function**				
GO:0005520	insulin-like growth factor binding	0.000	5.80	4
GO:0005201	extracellular matrix structural constituent	0.001	5.80	4
GO:0005515	protein binding	0.002	21.74	15
GO:0005344	oxygen transporter activity	0.002	4.35	3
GO:0019825	oxygen binding	0.005	4.35	3
GO:0000978	RNA polymerase II core promoter proximal region sequence-specific DNA binding	0.009	8.70	6
GO:0031720	haptoglobin binding	0.011	2.90	2
GO:0001077	transcriptional activator activity, RNA polymerase II core promoter proximal region sequence-specific bindin	0.014	7.25	5
GO:0005506	iron ion binding	0.038	5.80	4
GO:0048407	platelet-derived growth factor binding	0.040	2.90	2
**KEGG**				
rno05033	Nicotine addiction	0.001	5.80	4
rno04723	Retrograde endocannabinoid signaling	0.009	5.80	4
rno05143	African trypanosomiasis	0.011	4.35	3
rno05144	Malaria	0.024	4.35	3
rno05031	Amphetamine addiction	0.028	4.35	3

Up-regulated PPI Network

ID	Term	*p* -value	Ratio (%)	Count
**Biological Process**				
GO:0009612	response to mechanical stimulus	0.000	12.07	7
GO:0032355	response to estradiol	0.000	13.79	8
GO:0051591	response to cAMP	0.000	10.34	6
GO:0001558	regulation of cell growth	0.000	8.62	5
GO:0051384	response to glucocorticoid	0.000	10.34	6
GO:0042542	response to hydrogen peroxide	0.000	8.62	5
GO:0009416	response to light stimulus	0.000	6.90	4
GO:0030199	collagen fibril organization	0.000	6.90	4
GO:0007565	female pregnancy	0.000	8.62	5
GO:0007568	aging	0.000	12.07	7
GO:0001649	osteoblast differentiation	0.001	8.62	5
GO:0042060	wound healing	0.001	8.62	5
GO:0032870	cellular response to hormone stimulus	0.001	6.90	4
GO:0015671	oxygen transport	0.001	5.17	3
GO:0045444	fat cell differentiation	0.002	6.90	4
GO:0001503	ossification	0.004	6.90	4
GO:0042493	response to drug	0.006	12.07	7
GO:0044344	cellular response to fibroblast growth factor stimulus	0.008	5.17	3
GO:0043434	response to peptide hormone	0.008	6.90	4
GO:0006366	transcription from RNA polymerase II promoter	0.008	10.34	6
GO:0009314	response to radiation	0.012	5.17	3
GO:0071277	cellular response to calcium ion	0.012	5.17	3
GO:0071376	cellular response to corticotropin-releasing hormone stimulus	0.012	3.45	2
GO:0045944	positive regulation of transcription from RNA polymerase II promoter	0.014	15.52	9
GO:0032570	response to progesterone	0.015	5.17	3
GO:0032964	collagen biosynthetic process	0.019	3.45	2
GO:0048839	inner ear development	0.019	5.17	3
GO:0061469	regulation of type B pancreatic cell proliferation	0.022	3.45	2
GO:0051592	response to calcium ion	0.029	5.17	3
GO:0007267	cell-cell signaling	0.029	5.17	3
GO:0071333	cellular response to glucose stimulus	0.032	5.17	3
GO:0045893	positive regulation of transcription, DNA-templated	0.033	10.34	6
GO:0034097	response to cytokine	0.039	5.17	3
GO:0051291	protein heterooligomerization	0.039	5.17	3
GO:0045725	positive regulation of glycogen biosynthetic process	0.046	3.45	2
**Cellular Component**				
GO:0031012	extracellular matrix	0.000	15.52	9
GO:0005615	extracellular space	0.000	25.86	15
GO:0070062	extracellular exosome	0.000	34.48	20
GO:0005581	collagen trimer	0.001	6.90	4
GO:0005833	hemoglobin complex	0.001	5.17	3
GO:0005578	proteinaceous extracellular matrix	0.001	10.34	6
GO:0031838	haptoglobin-hemoglobin complex	0.012	3.45	2
GO:0005576	extracellular region	0.020	12.07	7
GO:0072562	blood microparticle	0.050	5.17	3
**Molecular Function**				
GO:0005520	insulin-like growth factor binding	0.000	6.90	4
GO:0005344	oxygen transporter activity	0.001	5.17	3
GO:0019825	oxygen binding	0.004	5.17	3
GO:0000978	RNA polymerase II core promoter proximal region sequence-specific DNA binding	0.004	10.34	6
GO:0001077	transcriptional activator activity, RNA polymerase II core promoter proximal region sequence-specific binding	0.008	8.62	5
GO:0005201	extracellular matrix structural constituent	0.009	5.17	3
GO:0031720	haptoglobin binding	0.009	3.45	2
GO:0005515	protein binding	0.024	18.97	11
GO:0005506	iron ion binding	0.025	6.90	4
GO:0048407	platelet-derived growth factor binding	0.034	3.45	2
GO:0004867	serine-type endopeptidase inhibitor activity	0.043	5.17	3
**KEGG**				
rno05143	African trypanosomiasis	0.008	5.17	3
rno05144	Malaria	0.018	5.17	3

Down-regulated PPI Netwokr

ID	Term	*p* -value	Ratio (%)	Count
**Biological Process**				
GO:0007268	chemical synaptic transmission	0.002	27.27	3
GO:0003407	neural retina development	0.007	18.18	2
GO:0050806	positive regulation of synaptic transmission	0.011	18.18	2
GO:0051965	positive regulation of synapse assembly	0.027	18.18	2
GO:1902476	chloride transmembrane transport	0.034	18.18	2
**Cellular Component**				
GO:0030054	cell junction	0.000	45.45	5
GO:0030672	synaptic vesicle membrane	0.000	27.27	3
GO:0008021	synaptic vesicle	0.001	27.27	3
GO:0045211	postsynaptic membrane	0.003	27.27	3
GO:0045202	synapse	0.007	27.27	3
GO:0060076	excitatory synapse	0.015	18.18	2
**Molecular Function**				
GO:0005515	protein binding	0.034	36.36	4
**KEGG**				
rno05033	Nicotine addiction	0.000	36.36	4
rno04723	Retrograde endocannabinoid signaling	0.000	36.36	4
rno04724	Glutamatergic synapse	0.001	27.27	3
rno04721	Synaptic vesicle cycle	0.032	18.18	2

Table S6. Functional enrichment analysis in PPI networks of the whisker follicle

Total PPI Network

ID	Term	*p*-value	Ratio (%)	Count
**Biological Process**				
GO:0006412	translation	0.000	8.97	20
GO:0015986	ATP synthesis coupled proton transport	0.000	3.14	7
GO:0006417	regulation of translation	0.000	3.14	7
GO:0046034	ATP metabolic process	0.000	2.69	6
GO:0014002	astrocyte development	0.001	1.79	4
GO:0032543	mitochondrial translation	0.003	1.79	4
GO:0009060	aerobic respiration	0.004	1.79	4
GO:1902600	hydrogen ion transmembrane transport	0.005	2.24	5
GO:0045471	response to ethanol	0.005	3.59	8
GO:0010951	negative regulation of endopeptidase activity	0.006	3.14	7
GO:0034101	erythrocyte homeostasis	0.006	1.35	3
GO:0018149	peptide cross-linking	0.006	1.79	4
GO:0098869	cellular oxidant detoxification	0.007	2.24	5
GO:0000028	ribosomal small subunit assembly	0.011	1.79	4
GO:0035584	calcium-mediated signaling using intracellular calcium source	0.014	1.35	3
GO:0021762	substantia nigra development	0.015	1.79	4
GO:0006122	mitochondrial electron transport, ubiquinol to cytochrome c	0.016	1.35	3
GO:0045600	positive regulation of fat cell differentiation	0.016	1.79	4
GO:0006954	inflammatory response	0.016	4.04	9
GO:0006413	translational initiation	0.019	1.79	4
GO:0006749	glutathione metabolic process	0.019	1.79	4
GO:0000302	response to reactive oxygen species	0.022	1.35	3
GO:0070488	neutrophil aggregation	0.022	0.90	2
GO:0035606	peptidyl-cysteine S-trans-nitrosylation	0.022	0.90	2
GO:0097421	liver regeneration	0.022	1.79	4
GO:0007568	aging	0.022	4.04	9
GO:0051291	protein heterooligomerization	0.024	2.24	5
GO:0030216	keratinocyte differentiation	0.028	1.79	4
GO:0001731	formation of translation preinitiation complex	0.028	1.35	3
GO:0010629	negative regulation of gene expression	0.029	3.14	7
GO:0045727	positive regulation of translation	0.033	1.79	4
GO:1990418	response to insulin-like growth factor stimulus	0.043	0.90	2
GO:0006776	vitamin A metabolic process	0.043	0.90	2
GO:0071407	cellular response to organic cyclic compound	0.044	2.24	5
GO:0000060	protein import into nucleus, translocation	0.045	1.35	3
GO:0031663	lipopolysaccharide-mediated signaling pathway	0.045	1.35	3
GO:2001244	positive regulation of intrinsic apoptotic signaling pathway	0.047	1.35	3
GO:0007569	cell aging	0.047	1.35	3
GO:0006915	apoptotic process	0.047	4.04	9
**Cellular Component**				
GO:0005739	mitochondrion	0.000	27.35	61
GO:0070062	extracellular exosome	0.000	28.25	63
GO:0022627	cytosolic small ribosomal subunit	0.000	5.38	12
GO:0005743	mitochondrial inner membrane	0.000	8.07	18
GO:0000276	mitochondrial proton-transporting ATP synthase complex, coupling factor F(o)	0.000	2.69	6
GO:0043209	myelin sheath	0.000	5.83	13
GO:0005753	mitochondrial proton-transporting ATP synthase complex	0.000	2.69	6
GO:0005840	ribosome	0.000	4.93	11
GO:0005737	cytoplasm	0.000	39.46	88
GO:0015935	small ribosomal subunit	0.000	2.24	5
GO:0022625	cytosolic large ribosomal subunit	0.001	4.04	9
GO:0005763	mitochondrial small ribosomal subunit	0.002	1.79	4
GO:0005925	focal adhesion	0.002	5.83	13
GO:0005730	nucleolus	0.002	8.97	20
GO:0005747	mitochondrial respiratory chain complex I	0.004	2.24	5
GO:0001533	cornified envelope	0.005	1.79	4
GO:0005750	mitochondrial respiratory chain complex III	0.008	1.35	3
GO:0030529	intracellular ribonucleoprotein complex	0.012	2.69	6
GO:0016282	eukaryotic 43S preinitiation complex	0.014	1.35	3
GO:0033290	eukaryotic 48S preinitiation complex	0.014	1.35	3
GO:0005852	eukaryotic translation initiation factor 3 complex	0.020	1.35	3
GO:0005759	mitochondrial matrix	0.036	2.69	6
GO:0005689	U12-type spliceosomal complex	0.041	1.35	3
GO:0005829	cytosol	0.041	12.11	27
GO:0090661	box H/ACA telomerase RNP complex	0.045	0.90	2
GO:0005758	mitochondrial intermembrane space	0.048	1.79	4
**Molecular Function**				

Up-regulated PPI Network

ID	Term	*p*-value	Ratio (%)	Count
**Biological Process**				
GO:0045944	positive regulation of transcription from RNA polymerase II promoter	0.007	16.98	9
GO:1990090	cellular response to nerve growth factor stimulus	0.009	5.66	3
GO:0030182	neuron differentiation	0.010	7.55	4
GO:0001570	vasculogenesis	0.015	5.66	3
GO:0045893	positive regulation of transcription, DNA-templated	0.021	11.32	6
GO:0010629	negative regulation of gene expression	0.021	7.55	4
GO:0021987	cerebral cortex development	0.022	5.66	3
GO:0030643	cellular phosphate ion homeostasis	0.025	3.77	2
GO:0008283	cell proliferation	0.027	7.55	4
GO:0042127	regulation of cell proliferation	0.028	7.55	4
GO:0010952	positive regulation of peptidase activity	0.028	3.77	2
GO:0010468	regulation of gene expression	0.028	7.55	4
GO:0009725	response to hormone	0.031	5.66	3
GO:0001501	skeletal system development	0.034	5.66	3
GO:0046622	positive regulation of organ growth	0.036	3.77	2
GO:0048715	negative regulation of oligodendrocyte differentiation	0.038	3.77	2
GO:0043627	response to estrogen	0.039	5.66	3
GO:0043410	positive regulation of MAPK cascade	0.040	5.66	3
GO:0032270	positive regulation of cellular protein metabolic process	0.041	3.77	2
GO:0045651	positive regulation of macrophage differentiation	0.041	3.77	2
GO:0006508	proteolysis	0.042	9.43	5
GO:0001701	in utero embryonic development	0.043	7.55	4
GO:0001649	osteoblast differentiation	0.044	5.66	3
GO:0009987	cellular process	0.044	3.77	2
GO:0007623	circadian rhythm	0.045	5.66	3
GO:0071407	cellular response to organic cyclic compound	0.046	5.66	3
GO:0001764	neuron migration	0.047	5.66	3
GO:0031641	regulation of myelination	0.049	3.77	2
**Cellular Component**				
GO:0005737	cytoplasm	0.000	56.60	30
GO:0070062	extracellular exosome	0.000	33.96	18
GO:0005829	cytosol	0.009	20.75	11
GO:0005654	nucleoplasm	0.042	18.87	10
**Molecular Function**				

Down-regulated PPI Netwokr

ID	Term	*p*-value	Ratio (%)	Count
**Biological Process**				
GO:0006412	translation	0.000	11.18	19
GO:0015986	ATP synthesis coupled proton transport	0.000	4.12	7
GO:0046034	ATP metabolic process	0.000	3.53	6
GO:0006417	regulation of translation	0.001	2.94	5
GO:0032543	mitochondrial translation	0.001	2.35	4
GO:0009060	aerobic respiration	0.002	2.35	4
GO:1902600	hydrogen ion transmembrane transport	0.002	2.94	5
GO:0034101	erythrocyte homeostasis	0.003	1.76	3
GO:0045471	response to ethanol	0.005	4.12	7
GO:0000028	ribosomal small subunit assembly	0.005	2.35	4
GO:0021762	substantia nigra development	0.007	2.35	4
GO:0014002	astrocyte development	0.008	1.76	3
GO:0006749	glutathione metabolic process	0.008	2.35	4
GO:0006122	mitochondrial electron transport, ubiquinol to cytochrome c	0.009	1.76	3
GO:0000302	response to reactive oxygen species	0.012	1.76	3
GO:0045727	positive regulation of translation	0.015	2.35	4
GO:0035606	peptidyl-cysteine S-trans-nitrosylation	0.016	1.18	2
GO:0070488	neutrophil aggregation	0.016	1.18	2
GO:0098869	cellular oxidant detoxification	0.018	2.35	4
GO:2001244	positive regulation of intrinsic apoptotic signaling pathway	0.027	1.76	3
GO:0034976	response to endoplasmic reticulum stress	0.028	2.35	4
GO:0006776	vitamin A metabolic process	0.032	1.18	2
GO:0018149	peptide cross-linking	0.032	1.76	3
GO:0002793	positive regulation of peptide secretion	0.040	1.18	2
GO:0000454	snoRNA guided rRNA pseudouridine synthesis	0.040	1.18	2
GO:0000398	mRNA splicing, via spliceosome	0.045	2.35	4
GO:0051291	protein heterooligomerization	0.047	2.35	4
GO:0018119	peptidyl-cysteine S-nitrosylation	0.047	1.18	2
**Cellular Component**				
GO:0005739	mitochondrion	0.000	32.35	55
GO:0022627	cytosolic small ribosomal subunit	0.000	7.06	12
GO:0005743	mitochondrial inner membrane	0.000	10.00	17
GO:0000276	mitochondrial proton-transporting ATP synthase complex, coupling factor F(o)	0.000	3.53	6
GO:0005840	ribosome	0.000	6.47	11
GO:0043209	myelin sheath	0.000	7.06	12
GO:0005753	mitochondrial proton-transporting ATP synthase complex	0.000	3.53	6
GO:0070062	extracellular exosome	0.000	26.47	45
GO:0022625	cytosolic large ribosomal subunit	0.000	5.29	9
GO:0015935	small ribosomal subunit	0.000	2.94	5
GO:0005730	nucleolus	0.000	11.18	19
GO:0005763	mitochondrial small ribosomal subunit	0.001	2.35	4
GO:0005747	mitochondrial respiratory chain complex I	0.001	2.94	5
GO:0005925	focal adhesion	0.002	6.47	11
GO:0005750	mitochondrial respiratory chain complex III	0.005	1.76	3
GO:0030529	intracellular ribonucleoprotein complex	0.021	2.94	5
GO:0005758	mitochondrial intermembrane space	0.024	2.35	4
GO:0005689	U12-type spliceosomal complex	0.024	1.76	3
GO:0005737	cytoplasm	0.028	34.12	58
GO:0001533	cornified envelope	0.028	1.76	3
GO:0071011	precatalytic spliceosome	0.031	1.76	3
GO:0005634	nucleus	0.032	31.76	54
GO:0090661	box H/ACA telomerase RNP complex	0.034	1.18	2
GO:1990726	Lsm1-7-Pat1 complex	0.043	1.18	2
GO:0031429	box H/ACA snoRNP complex	0.043	1.18	2
GO:0005762	mitochondrial large ribosomal subunit	0.045	1.76	3
**Molecular Function**				

GO:0003735	structural constituent of ribosome	0.000	12.11	27
GO:0044822	poly(A) RNA binding	0.000	17.94	40
GO:0046933	proton-transporting ATP synthase activity, rotational mechanism	0.000	2.24	5
GO:0015078	hydrogen ion transmembrane transporter activity	0.000	2.24	5
GO:0003723	RNA binding	0.002	6.28	14
GO:0008121	ubiquinol-cytochrome-c reductase activity	0.003	1.35	3
GO:0008137	NADH dehydrogenase (ubiquinone) activity	0.008	1.79	4
GO:0005515	protein binding	0.008	13.00	29
GO:0016209	antioxidant activity	0.015	1.35	3
GO:0005198	structural molecule activity	0.018	3.14	7
GO:0003743	translation initiation factor activity	0.026	1.79	4
GO:0031369	translation initiation factor binding	0.033	1.35	3
GO:0035662	Toll-like receptor 4 binding	0.043	0.90	2
GO:0002020	protease binding	0.046	2.24	5
GO:0016887	ATPase activity	0.047	2.69	6
**KEGG**				
rno03010	Ribosome	0.000	11.21	25
rno00190	Oxidative phosphorylation	0.000	7.62	17
rno05016	Huntington's disease	0.000	8.52	19
rno05010	Alzheimer's disease	0.000	7.62	17
rno05012	Parkinson's disease	0.000	6.73	15
rno04932	Non-alcoholic fatty liver disease (NAFLD)	0.000	4.93	11
rno03040	Spliceosome	0.013	3.14	7
rno04260	Cardiac muscle contraction	0.025	2.24	5

GO:0008022	protein C-terminus binding	0.023	7.55	4
GO:0019899	enzyme binding	0.025	9.43	5
GO:0016504	peptidase activator activity	0.028	3.77	2
GO:0042802	identical protein binding	0.038	11.32	6
**KEGG**				
rno05205	Proteoglycans in cancer	0.021	7.55	4
rno05215	Prostate cancer	0.028	5.66	3
rno04931	Insulin resistance	0.042	5.66	3

GO:0003735	structural constituent of ribosome	0.000	15.88	27
GO:0044822	poly(A) RNA binding	0.000	22.35	38
GO:0046933	proton-transporting ATP synthase activity, rotational mechanism	0.000	2.94	5
GO:0015078	hydrogen ion transmembrane transporter activity	0.000	2.94	5
GO:0008121	ubiquinol-cytochrome-c reductase activity	0.002	1.76	3
GO:0008137	NADH dehydrogenase (ubiquinone) activity	0.004	2.35	4
GO:0003723	RNA binding	0.004	6.47	11
GO:0005515	protein binding	0.016	12.94	22
GO:0031369	translation initiation factor binding	0.018	1.76	3
GO:0035662	Toll-like receptor 4 binding	0.032	1.18	2
GO:0034513	box H/ACA snoRNA binding	0.047	1.18	2
**KEGG**				
rno03010	Ribosome	0.000	14.71	25
rno00190	Oxidative phosphorylation	0.000	10.00	17
rno05016	Huntington's disease	0.000	10.59	18
rno05010	Alzheimer's disease	0.000	9.41	16
rno05012	Parkinson's disease	0.000	8.24	14
rno04932	Non-alcoholic fatty liver disease (NAFLD)	0.000	5.88	10
rno03040	Spliceosome	0.004	4.12	7
rno01100	Metabolic pathways	0.044	12.94	22

Table S7. KEGG pathway enrichment analysis of modules in the striatum PPI network.

**Module 1 (MCODE score = 5.167)**			
Kegg pathway	Kegg ID	No. of associated genes	*p*-value
Nicotine addiction	KEGG:05033	4	0.000
Retrograde endocannabinoid signaling	KEGG:04723	4	0.000
Amphetamine addiction Glutamatergic	KEGG:05031	3	0.000
synapse	KEGG:04724	3	0.000
Osteoclast differentiation	KEGG:04380	3	0.001
Cocaine addiction	KEGG:05030	2	0.002
**Module 2 (MCODE score = 4.667)**			
Kegg pathway	Kegg ID	No. of associated genes	*p*-value
Protein digestion and absorption	KEGG:04974	2	0.002
AGE-RAGE signaling pathway in diabetic complications	KEGG:04933	2	0.002
Amoebiasis	KEGG:05146	2	0.002
Relaxin signaling pathway	KEGG:04926	2	0.003
Platelet activation	KEGG:04611	2	0.003
**Module 3 (MCODE score = 2.857)**			
Kegg pathway	Kegg ID	No. of associated genes	*p*-value
African trypanosomiasis	KEGG:05143	3	0.000
Malaria	KEGG:05144	3	0.000
Prion diseases	KEGG:05020	2	0.000
Staphylococcus aureus infection	KEGG:05150	2	0.001
Pertussis	KEGG:05133	2	0.001
Complement and coagulation cascades Chagas	KEGG:04610	2	0.002
disease (American trypanosomiasis) Systemic	KEGG:05142	2	0.003
lupus erythematosus	KEGG:05322	2	0.004

Table S8. KEGG pathway enrichment analysis of modules in the whisker follicle PPI network.

**Module 1 (MCODE score = 17.924)**			
Kegg pathway	Kegg ID	No. of associated genes	*p*-value
Ribosome	KEGG:03010	24	0.000
Huntington disease Oxidative	KEGG:05016	10	0.000
phosphorylation Alzheimer	KEGG:00190	7	0.000
disease Parkinson disease	KEGG:05010	7	0.000
	KEGG:05012	6	0.000
**Module 2 (MCODE score = 15.857)**			
Kegg pathway	Kegg ID	No. of associated genes	*p*-value
Oxidative phosphorylation Thermogenesis	KEGG:00190	17	0.000
Alzheimer disease	KEGG:04714	17	0.000
Parkinson disease	KEGG:05010	15	0.000
Huntington disease	KEGG:05012	14	0.000
Non-alcoholic fatty liver disease (NAFLD)	KEGG:05016	15	0.000
Cardiac muscle contraction	KEGG:04932	9	0.000
Retrograde endocannabinoid signaling	KEGG:04260	4	0.000
Ribosome	KEGG:04723	5	0.000
	KEGG:03010	5	0.000
**Module 3 (MCODE score = 10.254)**			
Kegg pathway	Kegg ID	No. of associated genes	*p*-value
Spliceosome	KEGG:03040	7	0.000
Ribosome	KEGG:03010	5	0.000

Table S9. KEGG pathway enrichment analysis of 20 hub genes in the striatum and whisker follicle.

KEGG	KEGG ID	p-value	count	genes
Striatum				
Nicotine addiction	rno05033	0.000	3	[Slc17a6, Gria2, Slc17a7]
Amphetamine addiction	rno05031	0.001	3	[Gria2, Arc, Fosb]
Glutamatergic synapse	rno04724	0.002	3	[Slc17a6, Gria2, Slc17a7]
Retrograde endocannabinoid signaling	rno04723	0.003	3	[Slc17a6, Gria2, Slc17a7]
African trypanosomiasis	rno05143	0.004	2	[Hba2, LOC689064]
Cocaine addiction	rno05030	0.007	2	[Gria2, Fosb]
Malaria	rno05144	0.007	2	[Hba2, LOC689064]
Synaptic vesicle cycle	rno04721	0.008	2	[Slc17a6, Slc17a7]
Protein digestion and absorption	rno04974	0.015	2	[Col3a1, Col1a1]
Amoebiasis	rno05146	0.015	2	[Col3a1, Col1a1]
AGE-RAGE signaling pathway in diabetic complications	rno04933	0.015	2	[Col3a1, Col1a1]
Relaxin signaling pathway	rno04926	0.019	2	[Col3a1, Col1a1]
Platelet activation	rno04611	0.019	2	[Col3a1, Col1a1]
Osteoclast differentiation	rno04380	0.019	2	[Junb, Fosb]
Whisker follicle				
Alzheimer's disease	rno05010	0.001	4	[Ndufa2, Gapdh, App, Atp5e]
Huntington's disease	rno05016	0.028	3	[Sod1, Ndufa2, Atp5e]
Longevity regulating pathway - multiple species	rno04213	0.047	2	[Sod1, Akt1]

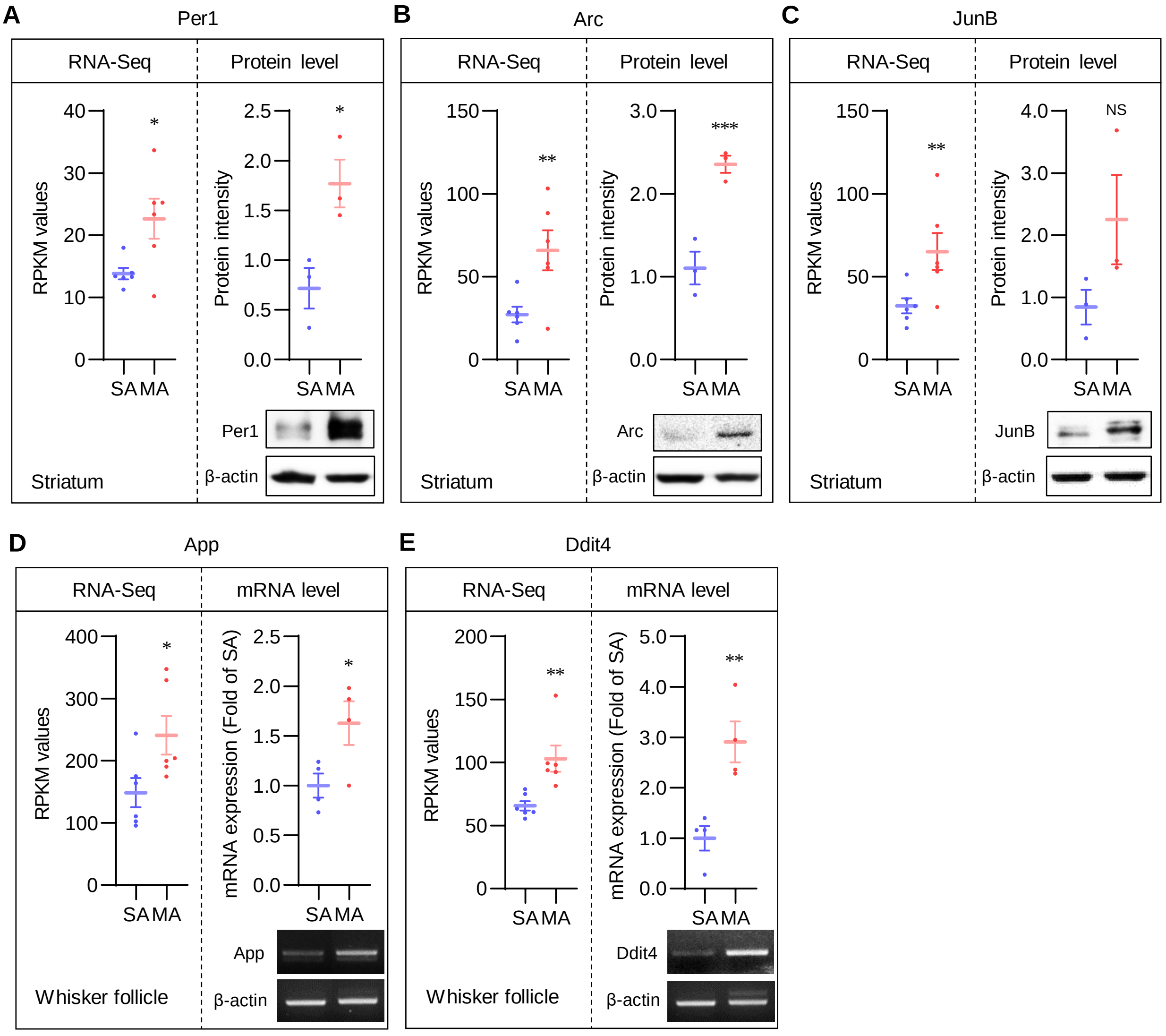

Figure S5. Validation of biomarkers in striatum and whisker follicle
